# Mitochondrial DNA and the Y chromosome suggest the settlement of Madagascar by Indonesian sea nomad populations

**DOI:** 10.1186/s12864-015-1394-7

**Published:** 2015-03-17

**Authors:** Pradiptajati Kusuma, Murray P Cox, Denis Pierron, Harilanto Razafindrazaka, Nicolas Brucato, Laure Tonasso, Helena Loa Suryadi, Thierry Letellier, Herawati Sudoyo, François-Xavier Ricaut

**Affiliations:** Laboratoire d’Anthropologie Moléculaire et Imagérie de Synthèse UMR-5288, Université de Toulouse, Toulouse, France; Statistics and Bioinformatics Group, Institute of Fundamental Sciences, Massey University, Palmerston North, New Zealand; Center for Linguistics, University of Leiden, Leiden, Netherlands; Genome Diversity and Diseases Laboratory, Eijkman Institute for Molecular Biology, Jakarta, Indonesia; Department of Medical Biology, Faculty of Medicine, University of Indonesia, Jakarta, Indonesia

**Keywords:** Ma’anyan, Indonesia, Madagascar, Sea nomad, Mitochondrial DNA, Y chromosome

## Abstract

**Background:**

Linguistic, cultural and genetic characteristics of the Malagasy suggest that both Africans and Island Southeast Asians were involved in the colonization of Madagascar. Populations from the Indonesian archipelago played an especially important role because linguistic evidence suggests that the Malagasy language branches from the Southeast Barito language family of southern Borneo, Indonesia, with the closest language spoken today by the Ma’anyan. To test for a genetic link between Malagasy and these linguistically related Indonesian populations, we studied the Ma’anyan and other Indonesian ethnic groups (including the sea nomad Bajo) that, from their historical and linguistic contexts, may be modern descendants of the populations that helped enact the settlement of Madagascar.

**Result:**

A combination of phylogeographic analysis of genetic distances, haplotype comparisons and inference of parental populations by linear optimization, using both maternal and paternal DNA lineages, suggests that Malagasy derive from multiple regional sources in Indonesia, with a focus on eastern Borneo, southern Sulawesi and the Lesser Sunda islands.

**Conclusion:**

Settlement may have been mediated by ancient sea nomad movements because the linguistically closest population, Ma’anyan, has only subtle genetic connections to Malagasy, whereas genetic links with other sea nomads are more strongly supported. Our data hint at a more complex scenario for the Indonesian settlement of Madagascar than has previously been recognized.

**Electronic supplementary material:**

The online version of this article (doi:10.1186/s12864-015-1394-7) contains supplementary material, which is available to authorized users.

## Background

Prior to the European colonial period, Austronesian-speaking populations were the most widespread of any language family [1,2]. While most groups speaking Austronesian languages moved eastward, settling the Pacific Ocean, others moved westward through the Indian Ocean, reaching eastern Africa and Madagascar. Dispersing halfway around the world within the past two millennia, the Austronesian expansion is often considered the last substantial wave of migration in human prehistory [3-5].

Despite considerable research on the eastward Austronesian expansion, there is little equivalent research on the western edge, leaving major issues unresolved regarding the settlement of Madagascar. Although the exact nature and route of this movement is largely unknown, linguistic and anthropological evidence indicates strong Indonesian influences, as recorded in the vocabulary and socio-cultural life of Malagasy, the modern people of Madagascar [6-9]. Linguistic research suggests that the Malagasy language is derived from Southeast Barito (SEB), a subgroup of Austronesian languages, and is most closely related to the language spoken by the land-locked forest-dweller Ma’anyan in central and southeastern Kalimantan (Borneo) [6,10-13], one indigenous language among 73 others spoken in Borneo [14]. However, there is evidence of word borrowings from a small number of Austronesian languages spoken on other Indonesian islands as well [15,16]. This probably reflects multiple Austronesian arrivals to Madagascar from about 700 AD onward (although earlier dates cannot be completely excluded). One hypothesis is that earlier movements were perhaps linked to Southeast Barito speakers, with later arrivals during the 12th-15th centuries connected instead to the Srivijaya and Majapahit kingdoms of Southeast Asia [17,18].

These linguistic findings are broadly supported by genetic studies, which emphasize the shared Indonesian and African genetic heritage of Malagasy. A recent study of genome-wide SNP data suggests that the western and central regions of Indonesia (Java/Borneo/Sulawesi) have the closest genetic connections with Malagasy [19]. This is in agreement with previous studies of uniparental markers (mtDNA and the Y chromosome), which found genetic affinity between Malagasy and western Indonesian populations [20,21]. A key lineage linking Indonesia and Madagascar is the Polynesian motif (a mitochondrial DNA haplogroup, B4a1a1, characterized by the polymorphisms A14022G, T16217C, A16247G and C16261T) [22]. More recently, it has been recognized that Malagasy carry specific point mutation variants (mtDNA nucleotides 1473 and 3423), which together have been termed the Malagasy motif [23]. This Malagasy version of the Polynesian motif is distributed throughout Madagascar with frequencies in specific ethnic groups ranging from 11-50% [21,23-25]. While still debated, this relatively homogenous distribution has been interpreted as supporting the first arrival of the Polynesian motif during an early phase of Madagascar’s settlement [26]. To date, the Malagasy motif has not been found in Indonesia [26], or anywhere else outside Madagascar. However, this may simply reflect the paucity of Indonesian populations available for study.

The westward Austronesian expansion was likely associated with trading activities of the Srivijaya empire, as suggested by the many Malay loanwords present in Malagasy [15,27,28], and this trade has been hypothesized to involve some sea nomad groups (i.e., the Orang Laut, Bajo and Bugis) [18,29,30]. This trading network was dominated by men, thus hinting at a potential male bias in the Indonesian contribution to Malagasy, in concordance with the standard matrilineal/matrilocal bias of traditional Austronesian society [31-34]. Contact between the Srivijaya empire and southeast Borneo may have stimulated the dispersal of Southeast Barito speakers to Madagascar – possibly at the same time as the dispersal of Sama-Bajaw speakers (a different subgroup of Barito languages) from the same area [35]. In this context, the Bajo are one sea nomad population of particular interest. Today, the Bajo live in several coastal communities around East Borneo, Sulawesi, the Lesser Sunda islands and the Maluku islands [36,37]. Because the Austronesian migration to Madagascar and the Sama-Bajaw dispersal may be interrelated, we compare genetic data from recent seafaring populations, such as the Bajo, with the more settled Malagasy.

Similarly, whether Barito populations such as the Ma’anyan, the closest linguistic siblings to modern Malagasy, share close genetic lineages with the Malagasy also remains unanswered. For the first time, we report genetic data for the Ma’anyan and the Lebbo’ (a population from Borneo with no presumed role in the settlement of Madagascar) to determine whether the Ma’anyan have an especially close genetic connection with Malagasy. We also include Bajo sea nomads from Sulawesi to determine whether there is a common genetic link based on their shared involvement in long distance maritime trading networks. A large data set of published and unpublished Indonesian populations is included for comparative analysis [38,39]. To investigate sex-specific genetic connections between Indonesia and Madagascar, we analyze both maternal (mtDNA) and paternal (Y chromosome) variation. We propose that the genetic connections of Malagasy to Indonesia are not restricted to Borneo, but instead include maternal and paternal lineages from a wide range of source populations from southern Sulawesi and the Lesser Sunda islands. We therefore propose that the settlement of Madagascar may have been mediated, at least in part, by sea nomad groups.

## Results

### Y chromosome and mitochondrial DNA classification

Based on analysis of 96 Y chromosome binary markers (Additional file 1: Table S1), the majority of men in the Ma’anyan, Lebbo’ and Bajo carry haplogroups previously found in Southeast Asia, particularly C*, K*, and O* (Table 1). Only a few individuals carry Y chromosomes belonging to Western Eurasian haplogroups: R* (M207) [40,41] was found in four Bajo individuals, R1a (M17) [42,43] was found in one Ma’anyan individual, while the western Eurasian haplogroups L1a (M76) and T1a (M70) [44,45] were found in one and two Bajo individuals, respectively. Indian haplogroup R*, which includes R1a, has previously been identified in Bali, Java, Borneo, and Mandar (Additional file 2: Table S2), and thus could conceivably have transited through Indonesia (as opposed to a direct connection), but T1a and L1a have not been identified to date in any Indonesian population.

**Table 1:** **Y chromosome haplogroup frequencies in the Ma’anyan, Lebbo’ and Bajo**

**Haplogroups**		**Lebbo’**	**Ma’anyan**	**Bajo**	**Geographic origin**
C*	C-RPS4Y*	0.1333	0.5222	0.0370	SEA
K*	K-M9*	0.0667	--	--	SEA
KxLT	K-M526*	0.1333	0.0222	0.2222	SEA
O1a	O-M119*	--	0.0222	0.0370	SEA
O1a1	O-P203	--	0.1000	--	SEA
O2a1	O-M95*	0.3333	0.0778	--	SEA
O3	O-M122*	--	0.0111	0.0370	SEA
C1c	C-M38*	--	--	0.2222	SEA
O1a2	O-M110	0.2667	0.0222	--	SEA
O2a1a	O-M88	--	0.0111	--	SEA
O3a2	O-P201*	0.0667	0.1778	0.0741	SEA
O3a2b	O-M7	--	--	0.0370	SEA
M1a	M-186	--	--	0.0741	SEA
P*	P-M45*	--	0.0222	--	SEA
R*	M-207*	--	--	0.1481	WE
R1a	R-M17	--	0.0111	--	WE
T1a	T-M70	--	--	0.0741	WE
L1a	L-M76	--	--	0.0370	WE

Note.

SEA: Southeast Asian origin; WE: Western Eurasian origin; and paragroups are indicated using a "*" (star) suffix.

On the mitochondrial DNA (Table 2), the frequency distributions of haplogroups found in the Ma’anyan, Lebbo’ and Bajo are broadly similar to, and consistent with, patterns of maternal lineages in Indonesia. Indeed, four main geographical/historical affiliations can be observed: mainland Asia, the Austronesian expansion, western Eurasia/India, and New Guinea. In brief, mainland Asian mtDNA haplogroups (such as B4c2, M73, M74, M12) are carried by a majority of individuals (64%), followed by haplogroups that have been putatively linked with an Austronesian expansion out of Taiwan (such as B4a1a1, M7c1a4, F1*, E*; 32%). The remaining lineages likely derive from India and west Eurasia, and were only observed among the Ma’anyan (M2, M5a4, and M35a, ranging in frequency from 0.6-1.9%). The presence of Indian and other western Eurasian genetic traces has been observed previously in Borneo, as well as Sumatra, Java and Bali [46,47] (Additional file 3: Table S3). Indian haplogroups are restricted to western Indonesia, particularly in regions historically involved in the ancient trading networks of the Hindu kingdoms (such as Srivijaya and Majapahit). Among the Bajo, we also observed the M1a Y haplogroup and Q1 mitochondrial haplogroup, which likely traces its ancestry to New Guinea or eastern Indonesia [39,48-50]. These haplogroups represent a trace of Papuan genetic input. This is perhaps due to the extensive trading network of the Bajo eastward to New Guinea [30] and/or earlier westward expansions of Papuan speakers from New Guinea to eastern Indonesia [51].

**Table 2:** **Mitochondrial haplogroup frequencies in the Ma’anyan, Lebbo’ and Bajo**

**Haplogroups**	**Lebbo’**	**Ma’anyan**	**Bajo**	**Geographic origin**
B4a	0.2105	0.0943	0.0741	MA
B4c1b	--	0.0252	0.0741	MA
B4c2	--	0.1887	--	MA
B5a	0.1579	0.0377	0.0370	MA
B4a2a	--	0.0440	--	MA
B4a4	--	--	0.0741	MA
B4b1	--	0.0818	--	MA
F3b1a	--	0.0189	--	MA
M12	--	0.0377	--	MA
M20	0.1579	0.0440	--	MA
M71a2	0.1579	--	--	MA
M73	--	0.0566	0.0370	MA
M74b1	--	0.1069	--	MA
N22	--	0.0189	--	MA
N9a6a	--	0.0252	--	MA
R22	--	--	0.0370	MA
R9b1a1a	0.1053	--	--	MA
X	--	--	0.0370	MA
Q1	--	--	0.0741	NG
B4a1a1	--	--	0.0370	Taiw
D4s	--	0.0629	--	Taiw
E1a	0.2105	--	0.0741	Taiw
F1a	--	0.0377	0.0370	Taiw
F1a1a	--	0.0377	--	Taiw
F1a3	--	--	0.0741	Taiw
F1a4	--	0.0252	--	Taiw
M7b1a1i	--	--	0.1481	Taiw
M7b1a2	--	0.0126	0.0741	Taiw
M7c1a4a	--	0.0126	0.1111	Taiw
M2	--	0.0063	--	WE
M35a	--	0.0189	--	WE
M5a4	--	0.0063	--	WE

Note.

MA: Mainland Asian origin; NG: New Guinea origin; Taiw: Out of Taiwan origin; WE: Western Eurasian origin.

### Paternal lineage proximity to Malagasy

#### Shared lineages

Among the haplogroups shared between Malagasy and Indonesians (Additional file 4: Table S4), four originated in Island Southeast Asia (C, O1a, O1a2, O2a1*), while six have western Eurasian origins (J1, J2, J2b, T*, L* and R1a). The Ma’anyan and five other Indonesian groups, all located around the Sulawesi sea (east Kalimantan Dayak, Java, Bali, Mandar and Sumba), share four of these Island Southeast Asian haplogroups. Importantly, Malagasy uniquely share just one subhaplogroup (O2a1a1-M88) with Ma’anyan, and this lineage has not been discovered in other regions of Indonesia. O2a1a1 may therefore be a marker of male genetic contributions from southern Borneo to Madagascar.

Shared Y chromosome haplogroups with a west Eurasian origin (J, T, L and R1a) (Additional file 4: Table S4) are also present in Indonesian populations, but only in the south and west of the Sulawesi sea. They occur at low frequency (<0.1%) in Java, Bali, Mandar and Bajo, but R1a is the only west Eurasian haplogroup identified in southern Borneo (Ma’anyan and east Kalimantan Dayak). However, west Eurasian lineages in Indonesia and Madagascar may result from independent dispersal events. Indeed, Indian and Arab traders have been active on both side of the Indian Ocean within the last three and one thousand years, respectively [47,52-56]. Therefore, west Eurasian haplogroups shared between Malagasy and Indonesians may have originated from Indonesia, or alternatively, they may have been obtained directly from southwestern Eurasia (the Middle East or India).

#### Population cross-comparisons

**Figure 1:**
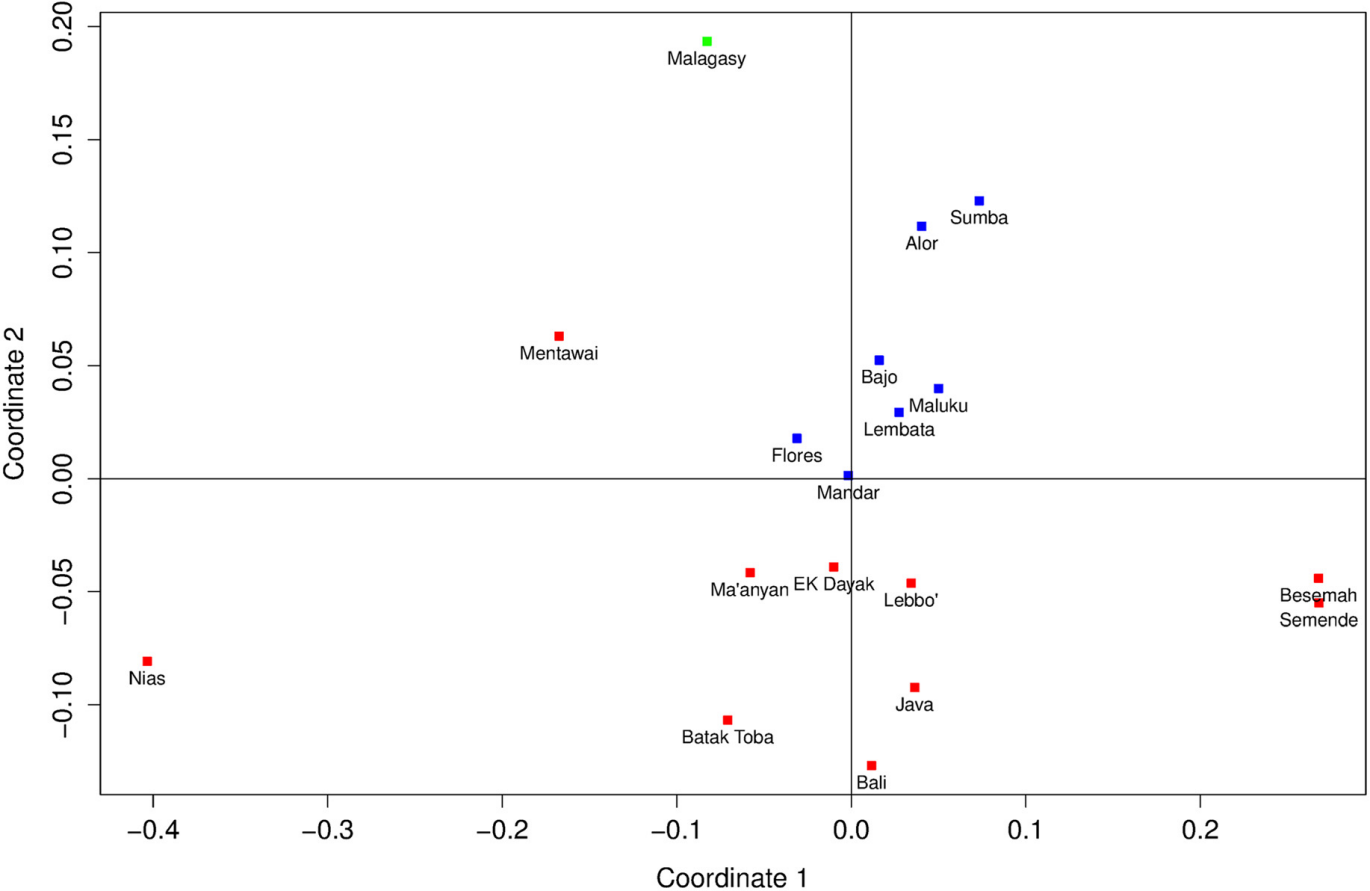
**MDS plot showing F**_**ST**_ **values between Indonesian and Malagasy populations based on Y chromosome haplogroup frequencies (Kruskal stress: 0.149).** Red: western Indonesians; blue: eastern Indonesians.

F_ST_ values based on Y chromosome haplogroup frequencies (Additional file 5: Table S5) were visualized on a multidimensional scaling (MDS) plot (Figure 1). Due to their statistically supported genetic homogeneity, Malagasy groups were pooled. The MDS plot (Figure 1) shows that Malagasy Y chromosome lineages are an outlier to Indonesian populations, in a similar way to certain Indonesian population outliers (Mentawai, Nias, Besemah, Semende). Y chromosome F_ST_ values between Malagasy and Indonesians are relatively high (F_ST_ > 0.2; Additional file 5: Table S5), mostly driven by the substantial African component of Malagasy (~65% of the paternal gene pool, but only ~30% of mtDNA [25,24]). No significant differences were observed to suggest specific genetic connections between Malagasy and eastern versus western Indonesians (Mann–Whitney U-test: P = 0.06). The Ma’anyan and other populations from Borneo cluster together with western Indonesian groups, including several population outliers (Mentawai, Nias, Besemah, Semende). The Bajo cluster with eastern Indonesian groups, consistent with the well-documented genetic division between western and eastern Indonesia broadly along the Wallace line.

**Figure 2:**
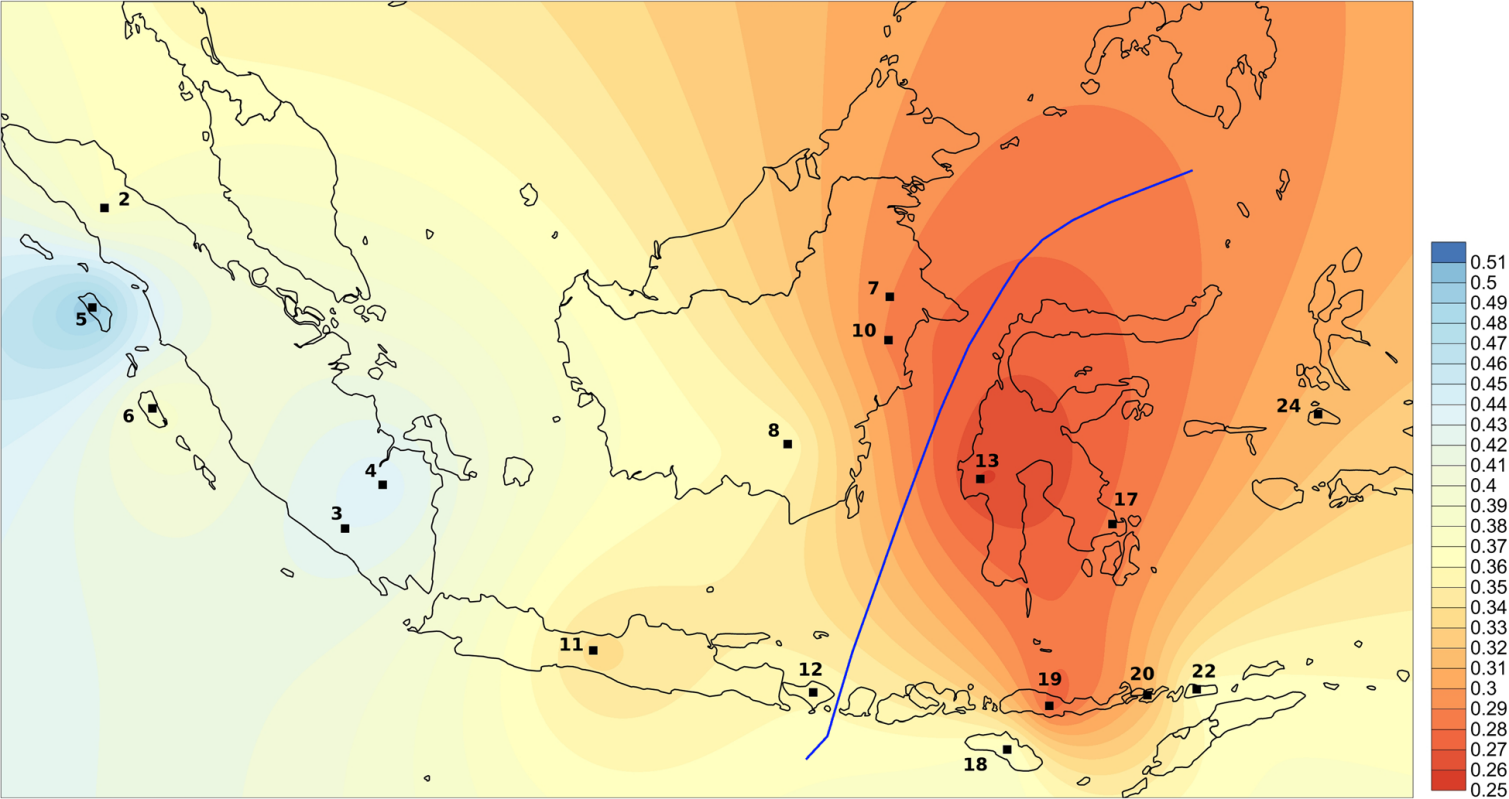
**Map of Y chromosome F**_**ST**_ **values obtained by pairwise comparison between Malagasy and Indonesian populations.** Dark red shading corresponds to lower pairwise F_ST_ values between Malagasy and Indonesian populations (represented by black squares), and dark blue to higher F_ST_ values. Note: 2. Batak Toba, 3. Besemah, 4. Semende, 5. Nias, 6. Mentawai, 7. Lebbo’, 8. Ma’anyan, 10. EK Dayak, 11. Java, 12. Bali, 13. Mandar, 17. Bajo, 18. Sumba, 19. Flores, 20. Lembata, 22. Alor, 24. Maluku.

When F_ST_ values are visualized with Surfer (Figure 2), the Indonesian populations with closest affinity to Malagasy (F_ST_ in the lower quartile of the range) are from regions near Wallace’s line in the west and south of the Sulawesi sea (southern Sulawesi, eastern Borneo and the Lesser Sunda islands). Populations with highest affinity to Malagasy are Mandar (Sulawesi), Flores (Lesser Sunda), Bajo (Sulawesi), and east Kalimantan Dayak and Lebbo’ (Borneo) (Additional file 5: Table S5). These results are supported by a linear optimization method, which aims to find the combination of Indonesian populations that most closely resembles the observed haplogroup diversity in Malagasy. This algorithm highlights two populations from the west and south of the Sulawesi sea, the Mandar (Sulawesi) and Lebbo’ (Borneo), as populations that produce a Y chromosome genetic profile most closely resembling the observed pattern, while still accounting for the predominantly African genetic background found in Malagasy (Additional file 6: Figure S1).

These geographical regions comprised part of the trading sphere of the Srivijaya empire, including several Javanese kingdoms that played a crucial role in the region: Heluodan (5th century), Tarumanagara (5th century), Walaing (Chinese Heling, 7th-8th centuries), Kahuripan/Kediri (11th century), Singasari (13th century) and Majapahit (13th -15th centuries) [57]. This region also hosted several houseboat nomad groups (such as the Bajo), which had ample opportunities to incorporate men from a wider regional watershed. The Ma’anyan from southern Borneo do not show any privileged link with Malagasy (indeed, they have a relatively high F_ST_ value showing genetic differentiation), despite being the only Indonesian population that shares Y haplogroup O2a1a with Malagasy. This may indicate that the genetic contribution of Ma’anyan was limited, either due to the recent arrival of this lineage in Ma’anyan, or perhaps O2a1a has since been lost or is still undetected in other Indonesian populations.

### Maternal lineage proximity to Malagasy

#### Shared lineages

Malagasy and Indonesians share mitochondrial haplogroups B4a1a, B4a1a1 (Polynesian motif), E1a1a, F3b, M7c1a4a, M32c and Q1 (Additional file 7: Table S6). Of these, B4a1a1, E1a1a and Q1 are found exclusively in eastern Indonesia. Conversely, F3b, B4a1a and M7c1a4a occur ubiquitously across both eastern and western Indonesia, and M32c has been observed in only one Javanese individual.

The Polynesian motif (B4a1a1) is considered strong evidence of Indonesian gene flow into Madagascar, where a variant is found at moderate frequency (11-50%). With the exception of Bali (Additional file 3: Table S3), B4a1a1 only occurs in eastern Indonesia. For the three new populations studied here, only the Bajo carry this Polynesian motif (just one of 27 individuals), and importantly, it was not found in any of our populations from Borneo. Furthermore, the specific Malagasy motif has not been found in Indonesia at all, including the new populations screened here. Considering the restricted geographic distribution of the Polynesian motif, it is most likely that this lineage from Madagascar traces back to eastern rather than western Indonesia.

**Figure 3:**
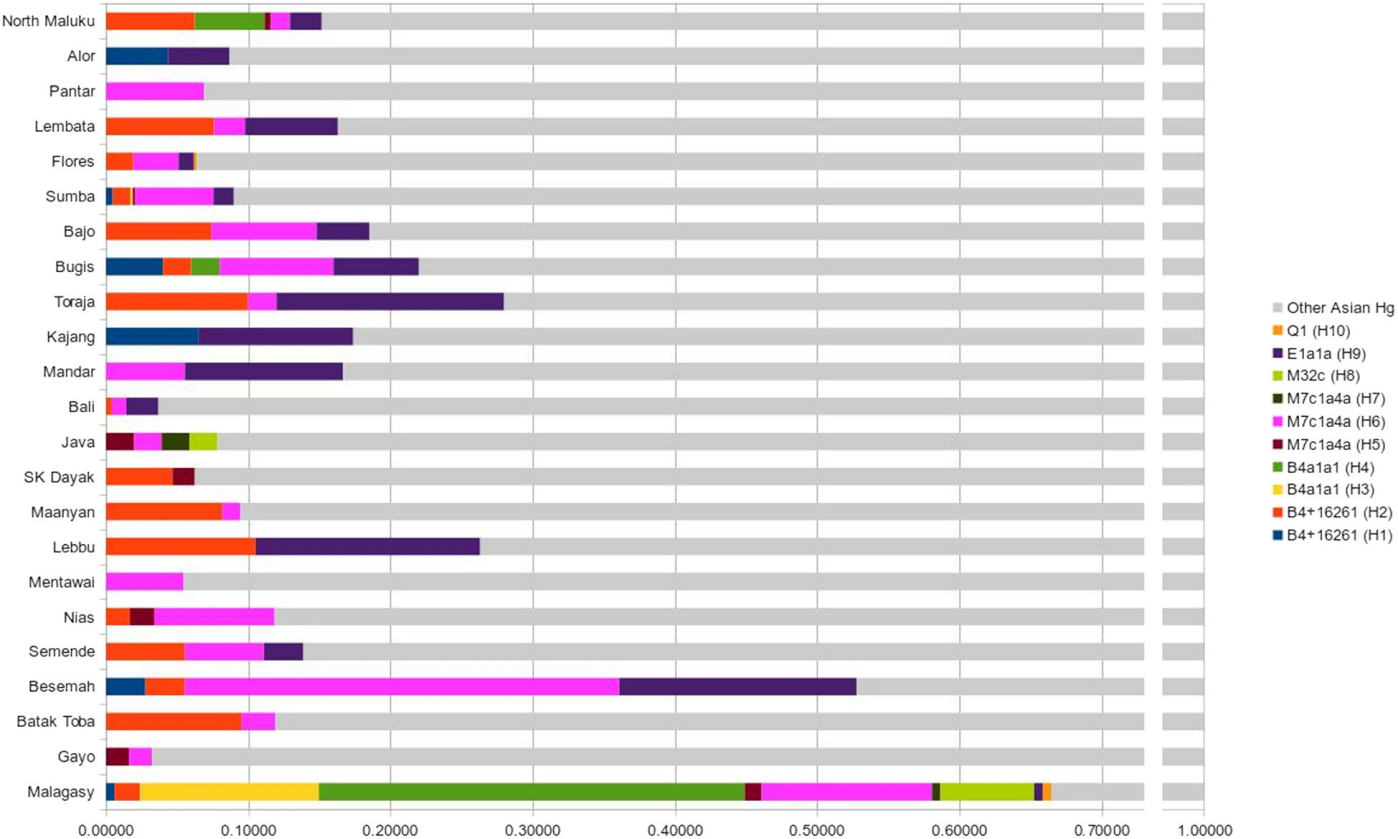
**Asian mtDNA haplotypes shared between Malagasy and Indonesian populations.**

Malagasy and Indonesians share ten haplotypes in seven haplogroups (Additional file 8: Table S7): two haplotypes each in B4a1a and B4a1a1; three haplotypes in M7c1a4a; and one haplotype in each of the other shared haplogroups. As shown in Figure 3, eastern Indonesian populations tend to share more haplotypes with Malagasy than western Indonesian groups. Populations from Sumba share the highest number of haplotypes (n = 6), followed by North Maluku and Sulawesi Bugis (n = 5). In a recurring theme, Ma’anyan exhibit limited sharing with only two haplotypes in common.

#### Population cross-comparisons

**Figure 4:**
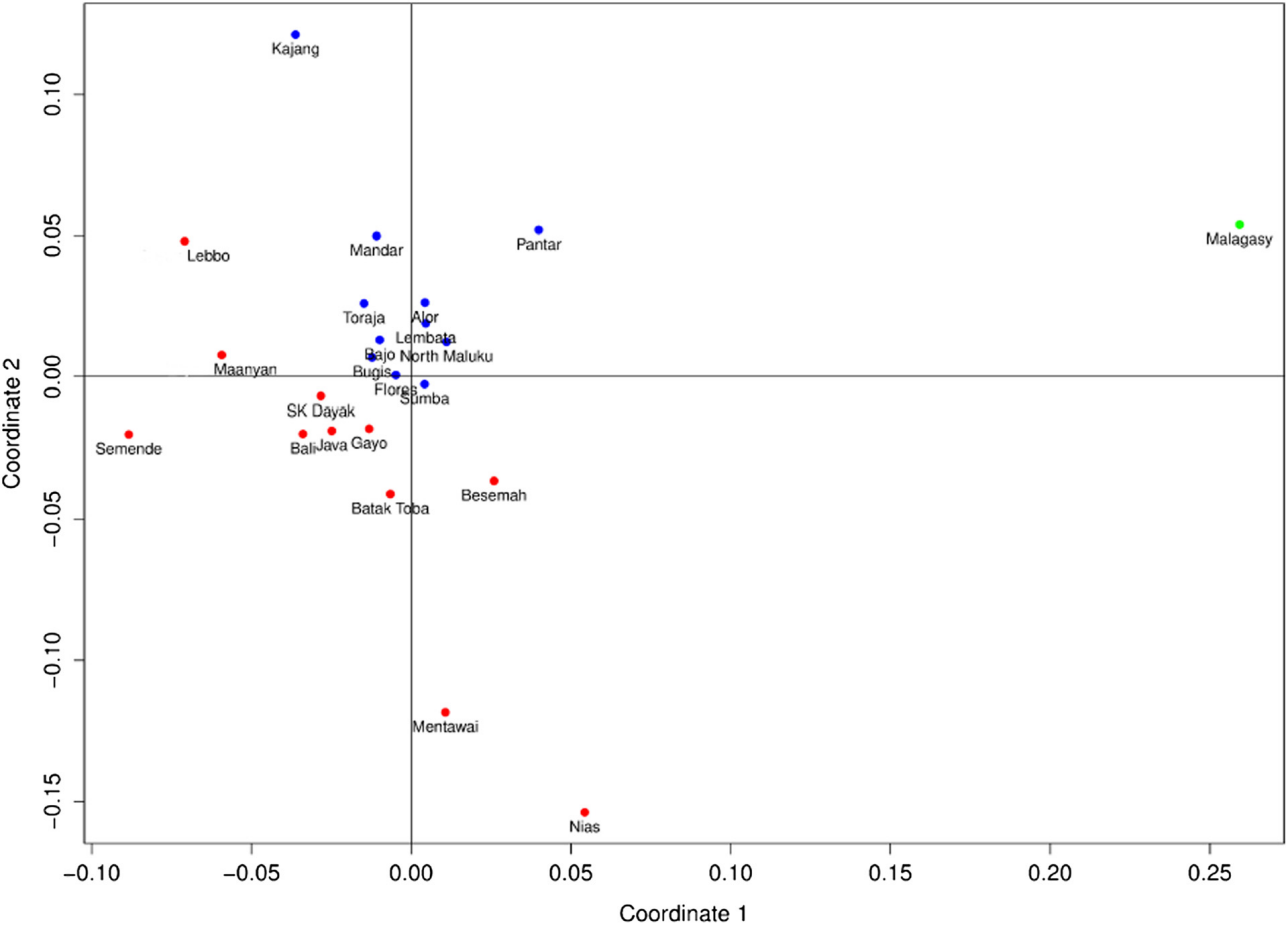
**MDS plot showing F**_**ST**_ **values between Indonesian and Malagasy populations based on mtDNA haplogroup frequencies (Kruskal stress: 0.143).** Red: western Indonesians; blue: eastern Indonesians.

**Figure 5:**
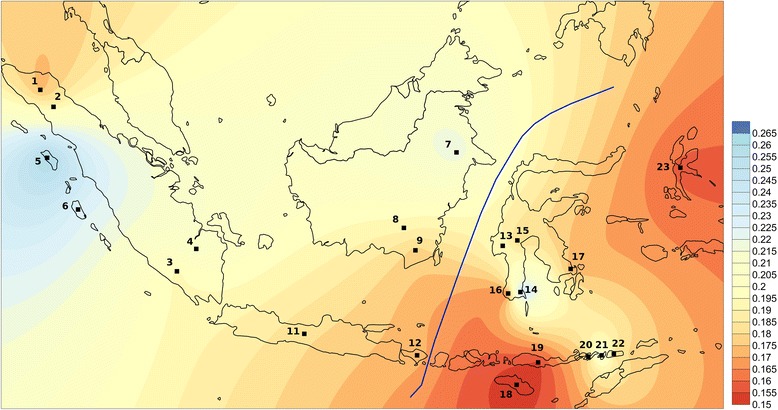
**Map of mitochondrial DNA F**_**ST**_ **values obtained by pairwise comparison between Malagasy and Indonesian populations.** Dark red shading corresponds to lower pairwise F_ST_ values between Malagasy and Indonesian populations (represented by black squares), and dark blue to higher F_ST_ values. Note: 1. Gayo, 2. Batak Toba, 3. Besemah, 4. Semende, 5. Nias, 6. Mentawai, 7. Lebbo’, 8. Ma’anyan, 9. SK Dayak, 11. Java, 12. Bali, 13. Mandar, 14. Kajang, 15. Toraja, 16. Bugis, 17. Bajo, 18. Sumba, 19. Flores, 20. Lembata, 21. Pantar, 22. Alor, 23. North Maluku.

The MDS plot (Figure 4, Malagasy groups again pooled), which is based on F_ST_ values from mtDNA haplogroup frequencies (Additional file 3: Table S3), shows that Malagasy maternal lineages differ markedly from those of Indonesians, while paternal lineages appear relatively closer (Figure 1). Unlike the Y chromosome data, which favors both eastern and western Indonesian sources, Malagasy are closer to the mtDNA diversity of eastern rather than western Indonesians (Mann–Whitney U test: P < 0.01). F_ST_ values (Additional file 9: Table S8) visualized in Surfer (Figure 5) show that the regions with closest affinity occur to the south and east of Sulawesi, and support an eastern Indonesian connection. Populations with higher affinity to Malagasy (F_ST_ in the lower quartile of the range) include Sumba and Flores, the Maluku islands and the Bugis of south Sulawesi. As seen on the Y chromosome, the Bajo cluster with eastern Indonesian groups, while the Ma’anyan and other populations from Borneo cluster with western Indonesian groups. Linear optimization results broadly agree with the F_ST_ results: Malagasy are most likely derived from a combination of eastern Indonesian groups, such as North Maluku, Bugis and Bajo (Additional file 10: Figure S2).

These multiple lines of genetic evidence suggest that Malagasy women may have originated predominantly from eastern Indonesia. Key pieces of evidence include the restricted distribution of the Polynesian motif in eastern Indonesia and patterns of shared maternal lineages. This is consistent with the hypothesis that Austronesians borrowed the Polynesian motif – which perhaps arose in the Bismarck archipelago – from indigenous sources in eastern Indonesia [58,59]. It is worth noting that eastern Indonesian influences occur in Madagascar: for cultivated plants (i.e., myths of origins, rituals for yams, the ancient importance of taro) [18,30], and through the influence of the Orang Laut language on some Malagasy dialects (particularly the Vezo) [11]. Eastern Indonesia had no recorded sea-faring cultures involved in long-distance trading, except for the Bugis and Bajo. However, maritime foraging and trade were likely more common in earlier millennia as networks of long-distance sea-based interactions have been in place since at least the early Holocene [60].

## Discussion

The Southeast Barito language subgroup includes two languages spoken by populations separated by the 7,500 km expanse of the Indian Ocean: the Malagasy of Madagascar, off the east coast of Africa, and the Ma’anyan of Borneo, an island in western Indonesia. Knowing this linguistic connection, we investigated genetic linkages between these two populations. Our results suggest that few genetic connections exist, either on the paternal Y chromosome or the maternal mtDNA. These results suggest that 1) the Ma’anyan groups sampled here are not directly related to the individuals who settled Madagascar, 2) subsequent demographic events have erased any genetic affinity between them, or 3) the Ma’anyan were just one population of many that contributed to the settlement of Madagascar (a possibility suggested by the exclusive sharing of Y chromosome haplogroup O2a1a). The first hypothesis might suggest that other Southeast Barito groups from southern Borneo (such as the Samihim or the Dusun Witu [61]) were involved instead.

These conclusions are drawn from a suite of complementary analyses, including phylogeography, haplotype sharing and linear optimization approaches. In combination, they paint a picture of the genetic dynamics between Indonesia and Madagascar. Although the geographic distribution of Indonesian populations that most closely reflect Malagasy genetic diversity are remarkably convergent for both male and female lineages, it is noticeable that the regions suggested by these two systems do not overlap perfectly. Our analyses suggest that populations from the south and west of the Sulawesi Sea (east Borneo, south Sulawesi and the Lesser Sunda islands) best explain Y chromosome diversity, while populations from the south and east of Sulawesi (south Sulawesi, the Lesser Sunda islands and the Maluku islands), all in eastern Indonesia, best explain mtDNA diversity. We emphasize that parts of these two regions overlap, thus potentially explaining both paternal and maternal affinity.

For instance, the distribution of shared lineages favors different source populations for maternal and paternal loci. Y chromosome haplogroup O2a1a is found only in Ma’anyan, while mtDNA haplogroups B4a1a1 and Q1 are found exclusively in eastern Indonesia. (Suggesting yet more connections, M32c has only been found in Java). F_ST_ and linear optimization results also highlight different source regions for the Y chromosome and mtDNA. Together, these patterns suggest that multiple regions may have contributed to the settlement of Madagascar, perhaps via one or a few admixed groups.

Sea nomads have been active traders along the eastern coast of Borneo, southern Sulawesi, the Lesser Sunda islands and the Maluku islands for at least the last few hundred years [62,63]. These mobile populations linked western and eastern Indonesia, and absorbed individuals from different regions. Sea nomads traveled with their families, even on long distance journeys [36,37]. Moreover, languages of the Sama-Bajaw group, as spoken by the sea nomad Bajo, form a subgroup of the Barito languages of southeast Kalimantan [35], although not the closest language subgroup to Malagasy. Their patterns of genetic diversity and lifestyle make them possible contenders for the Indonesian populations who helped enact the settlement of Madagascar, although a definite assignment remains elusive.

## Conclusion

We propose that the settlement of Madagascar had an Indonesian source location around southern Sulawesi, the Lesser Sunda islands and eastern Borneo. The populations involved may be related to modern sea nomad groups and the ancient Malay Srivijaya trading network. The Indonesian ancestors of Malagasy certainly carried maternal lineages with greater putative contributions from eastern Indonesia, and paternal lineages from both eastern and western Indonesia. The absence of any clear genetic connection between Malagasy and at least some populations speaking their most closely related language, Ma’anyan, raises important questions about the link between genes and language in the Indonesian dispersal across the Indian Ocean. Studying other Southeast Barito and sea nomad groups (such as the Orang Laut, who played a crucial role in the Srivijaya expansion) and the application of genome-wide genotyping technologies to provide additional genetic resolution promises to bring new insight to bear on these questions.

## Methods

### Population samples

All samples analyzed in this study were collected with informed consent from unrelated individuals. Subjects were surveyed for language affiliation, current residence, familial birthplaces, and a short genealogy of four generations to establish regional ancestry. A total of 205 DNA samples were analyzed from three ethnic groups: 159 Ma’anyan individuals were collected in Tamiang Layang (East Barito), Central Kalimantan, and Banjarmasin (South Kalimantan), representing the largest ethnically-defined population sample from Borneo to date; 19 Lebbo’ in East Kalimantan; and 27 sea nomad Bajo in Kendari (Sulawesi). Collection and use of these samples was approved by the Research Ethics Commissions at both the Eijkman Institute for Molecular Biology, Indonesia, and the University of Toulouse, France. We also included data for additional Indonesian populations from published [38,39,46] and unpublished sources (Gayo, North Maluku, and a mixed assemblage of other Dayak ethnic groups from the southern part of South Kalimantan province (“SK Dayak”) from the Eijkman Institute’s archived samples) (Additional file 11: Table S9). We also included published Malagasy data: seven Malagasy populations located in the southwest, southeast, and central highlands of Madagascar. The Malagasy were pooled for most analyses as these groups are genetically highly similar (between group F_ST_ < 0.05 and 95% of F_ST_ values non-significant (P > 0.05) for both mtDNA and the Y chromosome) [21,25,24]. In total, the mtDNA dataset comprises 529 Malagasy and 2,841 Indonesians, and the Y chromosome dataset comprises 371 Malagasy and 2,095 Indonesians.

### DNA extraction and genotyping

We collected blood samples for the Ma’anyan, except the Lebbo’ and Bajo, for which saliva samples were collected using the Oragene DNA Collection kit (http://dnagenotek.com). DNA was extracted from blood using a standard salting-out procedure, and from saliva using the manufacturer’s standard protocol. For paternal lineage analysis, 96 binary markers on the non-recombining region of the Y chromosome were analyzed. We used a nanofluidic dynamic array (Fluidigm, USA) high-throughput genotyping system. This system is developed for SNP genotyping assays and able to perform 9,216 real-time polymerase chain reactions (PCRs) (96 primers × 96 samples) on a single chip. The results were analyzed using the BioMark™ HD system (Fluidigm, USA) which integrated the Real-Time PCR Analysis software. Each haplogroup was assigned based on the updated ISOGG’s Y-DNA haplogroup tree [64] and the Y-Phylotree [65]. The full list of markers is shown in Additional file 1: Table S1. The mtDNA hypervariable region I was sequenced using primers F15989 (5’-CCCAAAGCTAAGATTCTAAT-3’) and R389 (5’-CTGGTTAGGCTGGTGTTAGG-3’). Sequences (GenBank accession numbers: KM590988-KM591192) were then analyzed and aligned against the revised Cambridge Reference Sequence (rCRS) [66] using the MAFFT aligner v.7 [67]. Mitochondrial haplogroups were determined with the Haplogrep program (http://haplogrep.uibk.ac.at) based on Phylotree v.16 [68]. The Malagasy motif, defined by mitochondrial coding region polymorphisms at nucleotides 1,473 and 3,423, were typed on all individuals carrying haplogroup B4a* using the method previously described [23].

### Statistical analysis

Pairwise F_ST_ distances between Indonesian and Malagasy populations were computed from haplogroup frequency data using Arlequin v.3.5 [69] with 5,040 permutations. Multidimensional scaling (MDS) from F_ST_ values based on Y chromosome and mitochondrial DNA haplogroup frequencies (Additional files 2 and 3: Table S2 and Table S3) was performed to visualize inter-population relations. The nonparametric Mann–Whitney U-test was applied to analyze the statistical significance of genetic affinity between Malagasy and Western/Eastern Indonesian groups. This phylogeographical division was defined by Wallace’s line, in agreement with previous human genetic population studies [39,70,71]. F_ST_ values obtained for the pairwise comparison of maternal and paternal lineages between Malagasy and Indonesian populations were plotted geographically with Surfer v.12.0 using the Kriging method. To determine which linear combination of Indonesian populations produces the closest genetic profile to that observed in Malagasy, we employed a statistical analysis of least squares with equalities and inequalities (lsei) algorithm in the R package, limSolve [72]. To capture sampling variance and drift dynamics, the genetic data were resampled 5,000 times and the linear optimization results visualized with box plots using the R package, ggplot. This analysis used mitochondrial DNA and Y chromosome haplogroup frequency distributions for both Malagasy and Indonesian populations (Additional files 2 and 3: Table S2 and Table S3). An African reference group was used to represent the non-Asian contribution to Malagasy. This reference comprised African samples from populations in North-, East-, Central- and South Africa [73-79]. Sharing of mitochondrial haplotypes was ascertained using Arlequin v.3.5 [69]. For this analysis, sequences from Tofanelli [21] were excluded due to their short length (360 bp compared to 520 bp for the present study).

## Additional files

Additional file 1: Table S1. List of Y chromosome SNP markers screened. Additional file 2: Table S2. Y chromosome haplogroup frequencies in Indonesian and Malagasy populations. Additional file 3: Table S3. Mitochondrial haplogroup frequencies in Indonesian and Malagasy populations. Additional file 4: Table S4. Y chromosome haplogroup sharing frequencies between Malagasy and Indonesian populations. Additional file 5: Table S5. Y chromosome F_ST_ distances between Malagasy and Indonesian populations. Additional file 6: Figure S1. Population proportion based on Y chromosome haplogroups estimated with the lsei algorithm. Note: black dots represent mean values. Additional file 7: Table S6. Mitochondrial haplogroup sharing frequencies between Malagasy and Indonesian populations. Additional file 8: Table S7. Mitochondrial haplotype sharing between Malagasy and Indonesian populations. Additional file 9: Table S8. Mitochondrial DNA F_ST_ distances between Malagasy and Indonesian populations. Additional file 10: Figure S2. Population proportion based on mitochondrial DNA haplogroups estimated with the lsei algorithm. Note: black dots represent mean values. Additional file 11: Table S9. List of populations used in this study.

## Abbreviations

SEB: Southeast Barito
SNP: Single nucleotide polymorphism
mtDNA: mitochondrial DNA
MDS: Multidimensional scaling
SK Dayak: South Kalimantan Dayak
EK Dayak: East Kalimantan Dayak
lsei: Least squares with equalities and inequalities
